# The role of extracellular vesicles in chronic lung allograft dysfunction and response to extracorporeal photopheresis

**DOI:** 10.1016/j.jhlto.2025.100322

**Published:** 2025-06-15

**Authors:** Rachel E. Crossland, Steven J. Bolton, Andrew J. Fisher

**Affiliations:** Translational and Clinical Research Institute, Faculty of Medical Science, Newcastle University, UK

**Keywords:** Extracellular vesicles, Chronic lung allograft dysfunction, Lung transplantation, Extracorporeal photopheresis, Immunomodulation

## Abstract

Current research highlights the growing role of extracellular vesicles (EV) in mechanisms of lung allograft dysfunction. In particular, EVs are involved in antigen presentation, where they are released from lung allografts and express tissue associated antigens which are recognized by recipient immune cells, thereby triggering an immune response against the transplanted lung. In the context of chronic rejection, patients with chronic lung allograft dysfunction (CLAD) demonstrate elevated levels of EVs, which contain diverse molecular cargo that can influence the alloimmune response. This highlights the potential of EVs as translatable biomarkers for the early detection, prediction, or diagnosis of lung allograft dysfunction. The mechanisms by which EVs contribute to this process may include immune cell activation, epithelial-to-mesenchymal transition, and disruption of angiogenesis. Furthermore, their immunomodulatory potential is evident by their emerging involvement in regulating the immune response during extracorporeal photopheresis (ECP) therapy following lung transplantation, where they contribute to the balance of immunoregulatory and autoimmune responses within a highly interwoven network. While ECP shows promise for broader or earlier use in solid organ transplantation, its application is limited by a lack of mechanistic understanding. This review summarizes the role of EVs in development of lung allograft dysfunction, their involvement in immunomodulation, and the current literature exploring their potential role in the mechanisms of ECP therapy.

## Highlights

• EVs express tissue antigens from lung allografts that may trigger immune responses in rejection.
• CLAD patients show elevated EV levels with distinct microRNA signatures and self-antigens.
• EVs show potential as biomarkers for early CLAD detection up to 12 months before formal diagnosis.
• ECP therapy's immunomodulatory effects may involve specific EV microRNA expression changes.
• Further studies needed to clarify EV's role in both CLAD pathogenesis and ECP mechanisms.

## Graphical abstract

**Figure:**
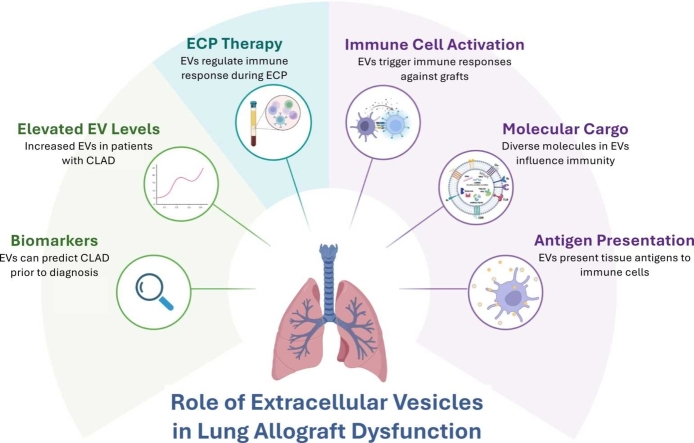

## Chronic lung allograft dysfunction

Lung transplantation (LTx) offers improved survival and quality of life to patients with life threatening chronic lung disease. Approximately 5000 LTxs are performed globally each year, however, data from the International Society for Heart and Lung Transplantation (ISHLT) registry show the median survival after LTx is just 6 years.^1^ This is mainly due to the development of progressive immune-mediated damage to the lung allograft, despite the use of immunosuppression, termed chronic lung allograft dysfunction (CLAD).^2^ This damage causes chronic inflammation and fibrosis in two patterns; either around the airways causing airway obstruction, known as bronchiolitis obliterans syndrome (BOS), or within the lung parenchyma affecting alveolar structure, known as restrictive allograft syndrome (RAS). CLAD is the most common cause of death after the first post-transplant year, meaning >50% of recipients develop CLAD within 5 years post-LTx. CLAD can progress rapidly, causing lung function decline, respiratory failure, and death. It often returns recipients to levels of disability and mortality risk they experienced immediately before transplant.^3^

The immunopathology of CLAD is complex, involving both innate and adaptive responses, alongside the immune cell secretome including cytokines, chemokines and proteases associated with tissue remodeling.^4^ Neutrophils, eosinophils, cytotoxic and T-helper cells are all known to contribute to development of CLAD. Eosinophils have been more specifically related to the RAS phenotype, which also demonstrates a more prominent humoral immune involvement with increased B-cells, immunoglobulins and complement deposition.^4^ Despite recent research leading to a better understanding of how the immune system contributes to the development of CLAD, the exact pathophysiological mechanisms are still not completely understood. However, it is believed to be the end-stage of a disease continuum marked by repeated lung injury, immune activation, tissue remodeling and repair, leading to irreversible fibrosis and ultimately allograft failure.^5^

### Extracellular vesicles and their role in antigen presentation and immune modulation

Extracellular vesicles (EV) have stimulated excitement in the scientific community due to their novel role in intercellular communication. Small EVs are a heterogeneous group of membrane-bound vesicles (50–150 nm in diameter), that include exosomes deriving from the internal endosomal system and microvesicles that bud from the plasma membrane. These cell-derived nanoparticles are released from nearly all cell types and form an essential component of an ancient intercellular communication system.^6^ EVs contain complex molecular information from their parent cell, and mediate local and systemic cellular communication by transferring their genetic and proteomic cargo.^6^ They are present in biofluids and have demonstrated enticing promise for non-invasive ‘Liquid Biopsies’. Thus, EVs have emerged as a novel class of potentially powerful biomarkers.

EVs play an essential role in control of the immune system, where their molecular cargo constantly balances immunoregulatory and autoimmune responses of surrounding cells in a highly interwoven network, to both initiate and regulate the immune response.^7^, ^8^ It has been shown that EVs can participate in antigen presentation though “cross-dressing”, whereby they transfer MHC-peptide complexes from professional APCs to non-professional APCs. Additionally, EVs can act via cross-presentation, where donor-derived antigens within EVs are processed and presented on MHC class I molecules to activate cytotoxic T lymphocytes**.**^8^ Thus, in transplantation, EVs can enhance the indirect pathway of allorecognition, by carrying donor-derived MHC molecules or processed donor peptides, which recipient APCs can internalize and present to T-cells, initiating immune responses against the allograft, and contributing to both acute and chronic rejection.

EVs can also modulate immune responses through functional interactions with dendritic cells (DCs). They can influence DC maturation and function by directly transferring surface molecules (e.g. PD-L1), delivering regulatory microRNAs, and altering cytokine secretion to promote a tolerogenic profile.^9^ EV-conditioned DCs exhibit reduced expression of co-stimulatory molecules (e.g. CD80) and enhanced secretion of anti-inflammatory cytokines such as IL-10, thus favoring Treg induction and suppression of effector T cells.^8^, ^9^ Importantly, EVs can establish feedback loops, that extend immune regulation across broader immune populations.

Furthermore, EVs derived from distinct immune cell subsets can exert differential effects that fine-tune immune responses. For example, EVs from activated immune cells can carry pro-inflammatory signals, while EVs from regulatory populations, such as Tregs, may be enriched in immunosuppressive factors that promote tolerance.^10^ Through these mechanisms, EVs influence the maturation, polarization, and differentiation of multiple immune cells, including T cells, monocytes and NK cells. Encapsulation and stabilization of EV-associated cytokines further extends the functional capacity of immune EVs, by regulating cytokine release and downstream proliferation, adding further complexity to the role of EVs in immune crosstalk.^7^

In the context of solid organ transplantation (SOT), EVs assist with communication between donor organ cells and recipient immune cells, and may inform on the function of an allograft.^11^ Thus, there is much interest in the use of EVs for preoperative assessment of organs, early postoperative monitoring of graft function, or the diagnosis of rejection, infection, ischemia-reperfusion injury and drug toxicity.^11^ Importantly, advances in EV research are not only enhancing diagnostic capabilities, but may also open therapeutic opportunities for modulating EV production, composition, or function to promote tolerance induction and potentially reduce the need for chronic immunosuppression^49^.

### Extracellular vesicles and lung allograft dysfunction

The immunological role of EVs in allograft rejection and tolerance is an area of increasing focus, including in the context of CLAD. Many questions remain, including the precise nature of the EVs themselves, the phenotype of EV-producing cells, and the mechanisms by which they influence alloimmunity.^12^

In 2015, Gregson *et al* hypothesized that mRNA derived from EVs may represent the bronchoalveolar lavage fluid (BALF) transcriptome and provide insights into allograft function during rejection.^13^ The group assessed the transcriptomics of BALF EVs, isolated by ultracentrifugation, from six LTxR with acute rejection (AR), and six without AR, using RNA sequencing (RNA-Seq). They showed that EVs from AR patients demonstrated a skewed inflammatory response, affecting both innate and adaptive immune systems. Gene set enrichment analysis identified an inflammatory environment with both MHC class I and II represented (Table 1). These results demonstrated the potential of EVs in a LTx context to provide crucial information on the underlying pathology of rejection in the lung allograft.

**Table 1:** Summary of Studies Assessing EVs During Lung Allograft Dysfunction Following Lung Transplantation. AR = acute rejection; Av. = average; BALF = bronchoalveolar lavage fluid; BOS = bronchiolitis obliterans syndrome; CF = cystic fibrosis; CLAD = chronic lung allograft dysfunction; DLTx = double lung transplantation; DSA = donor specific antibodies; Dx = diagnosis; ELISA = enzyme-linked immunoassay; EV = extracellular vesicles; F = female; FC = flow cytometry; LTx = lung transplantation; LTxR = lung transplantation recipient; M = male; N/A = not applicable; NK = not known; PGD = primary graft dysfunction; PR = precipitation reagent; RT-PCR = real-time polymerase chain reaction; RAS = restrictive allograft syndrome; RVI = respiratory viral infections; SAg = surface antigen; SC = sucrose cushion; SC-UC = sucrose cushion ultracentrifugation; SLTx = single lung transplantation; TEM = transmission electron microscopy; UC = ultracentrifugation; WB = western blot. Information taken from^13^, ^14^, ^15^, ^16^, ^17^, ^18^, ^19^, ^20^, ^21^, ^22^, ^23^

Author, Year	Disease	Controls	Study Group	Patient Gender	Patient Age	Tx	Timepoint of Samples	EV Isolation	EV Characterization	EV Source	EV Analysis & Technique	Overall EV Findings
Gregson et al, 2015^13^	AR	N/A	6 AR, 6 no AR	5 M 7 F	Av. 56 (range 39−72)	6 DLTx, 6 SLTx		UC	FC for CD9/CD63/CD81	BALF	RNA isolation using miRNeasy, RNA-Seq using Illumina HiSEquation 2500	AR samples were skewed toward an inflammatory response involving pathways in both the innate and adaptive immune systems.
Gunasekaran et al, 2017^14^	BOS, AR	Stable	10 BOS, 10 AR, 10 stable	21 M 9 F	Av. 57	All DLTx	1, 3, 6 and 12 months post LTx	BALF EVs: UC, Serum EVs: PR. All purified by SC-UC	TEM, WB for Annexin-V, serum EVs by FC for CD63	BALF and Serum	WB and FC for SAg. RNA isolation using mirVana miRNA isolation kit, microRNA profiling using Affymetrix microarray, individual microRNAs using TaqMan RT-PCR.	Donor HLA and SAgs were detected on EVs from LTxRs with AR and BOS, but not from stable LTxRs. EVs isolated from LTxRs with AR or BOS also contained immunoregulatory miRNAs.
Sharma et al, 2018^15^	BOS	Stable	20 BOS, 10 stable	23 M 7 F	Av. 52	30 LTxRs	NK	UC then SC	TEM, WB for CD9	Serum	WB for SAgs	LTxR diagnosed with BOS had EVs with higher expression of Col-V and Kα1T compared to stable patients.
Mohanakumar et al, 2019^16^	DSA BOS PGD AR RVI	Stable/ control	5 DSA 10 BOS 5 PGD 10 AR 15 RVI, 45 stable/ control	62 M 28 F	Av. 53	All DLTx	Average 25 months post LTx	Plasma EVs PR, culture media EVs UC	WB for CD9	Plasma and culture media	ELISA for donor HLA, WB for SAg, costimulatory molecules, transcription factors, adhesion molecules, 20 s proteosome and ER stress markers.	Patients with PGD, RVI, AR and DSA had EVs containing SAg; EVs from stable recipients did not. EVs from recipients with PGD, AR, and DSA had CD80, CD86, MHC-II, transcription factor, and 20S proteasome.
Gunasekaran et al, 2018^17^	BOS	Stable	10 BOS, 10 stable	10 M 10 F	Av. 52	20 DLTx	NK	UC then SC	NK	Serum	WB for co-stimulatory molecules, SAg, transcription factors and adhesion molecules.	SAg (Kα1T and Col-V), MHC class II, CD40, CD80, CD86, and transcription factors class II MHC trans-activator, NF-κB, HIF1-α, IL−1R–associated kinase 1, MyD88, and 20S proteasome were detected in EVs from BOS, but not stable LTxR. Adhesion molecules were present in both groups. C57BL/6 mice immunized with EVs from BOS but not stable LTxR demonstrated Ab to SAg (Col-V, Kα1T, and HLA). Reduced IL−10–producing cells were seen in BOS EV immunized mice compared with mice immunized with stable EVs.
Sharma et al, 2020^18^	BOS	Stable	Pediatric: 6 BOS, 13 stable	13 M 6 F	Av. 13	NK	1, 6 and 12 month	UC then SC	Nanosight for size and quantification, WB for TSG1, CD9 and Alix	Plasma	ELISA for HLA-A2, WB for SAg, costimulatory molecules, transcription factors, MHC-II, adhesion molecules and 20S proteosome.	EVs from BOS LTxRs had increased SAgs, donor HLA class I, MHC-II, transcription factors, co-stimulatory molecules, and 20S proteasome compared to stable. EVs were detected in the circulation before BOS. EVs from BOS were distinct in inducing humoral and cellular responses to SAgs. SAgs were detected on circulatory EVs 12 months before BOS diagnosis.
Sharma et al, 2020^19^	BOS	Stable	41 BOS, 30 control/ stable	22 M 9 F	Av. 52	All DLTx	6 and 12 month prior to BOS, time of BOS	UC, or PR when plasma was <100ul	Nanosight for size and quantification, WB for CD9 and Alix	Plasma	WB for SAgs Ka1T and Col-V	EVs from LTxRs with BOS showed increased SAgs (Kα1T and Col-V) 12 months before diagnosis (100% specificity, 90% sensitivity).
Ravichandran et al, 2025^20^	CLAD	Stable	25 CLAD, 32 stable (CF LTx). 21 CLAD, 21 stable (non-CF LTx).	54 M 45 F	Av. 39	78 DLTx, 21 SLTx	At CLAD, 6 and 12 month pre-CLAD	PR followed by filtration	TEM for CD9, ExoView for CD9, CD63 and CD81.	Plasma	WB for SAg Col-V and Ka1T, and LKB1, ExoView for LKB1, CD63, CD81 and CD9. TEM for co-localization of LKB1, LC-IgG and CD9.	EVs from non-CF LTRs had higher SAg and lower LKB1 12 months before CLAD, compared to stable LTRs. In CF LTRs, only LKB1 levels were lower 6 months before Dx. EVs 6 months before CLAD in CF LTRs also had lower LKB1 and LKB1/CD9 particles.
Rahman et al, 2022^21^	PGD	Stable	15 PGD, 15 stable	20 M 10 F	Av. 54	All DLTx	Pre-LTX and at PGD	PR followed by filtration	Nanosight for size and quantification, WB for CD9	Plasma	WB for LKB1, Col-V, Ka1T, NF-kB, E-Cadherin, vimentin and α-SMA	LKB1 was lower in PGD EVs compared to no-PGD. Within PGD, lower post-LTx LKB1 was associated with CLAD development.
Bansal et al, 2022^22^	BOS, RAS	Stable	18 BOS, 13 RAS, 5 stable	20 M, 16 F	Av. 58	35 DLTx, 1 SLTX	At time of BOS/RAS diagnosis	UC followed by filtration	Nanosight for size and quantification	Plasma	WB for Col-V, Ka1T, 20Sa3, HLA-I, HLA-DQ, HLA-DR, NFkB, CIITA, PIGR. NanoString for microRNAs. ELISA for SAg Col-V and Ka1T. Luminex for HLA Abs,	CIITA, NFkB, polymeric immunoglobulin receptor protein, 20S proteasome, HLA-DQ, and HLA-DR were higher in RAS EVs than BOS EVs. RAS plasma had distinct EV miRNA. Immunization of C57BL/6 mice with RAS EVs showed severe inflammation and peribronchial fibrosis, whereas BOS EVs induced patchy inflammation and fibrosis.
Kawana et al, 2024^23^	CLAD	Stable	24 CLAD, 35 stable	NK	NK	NK	At CLAD Dx and 12 month prior	NK	NK	Plasma	MiRNA array for microRNA expression profiling. RT-PCR for specific microRNA expression.	Plasma EV levels of miR−17−5p and miR−150−5p were higher in CLAD vs. non-CLAD at Dx. ROC analysis showed both miRNAs discriminated CLAD from non-CLAD.

**Figure 1:**
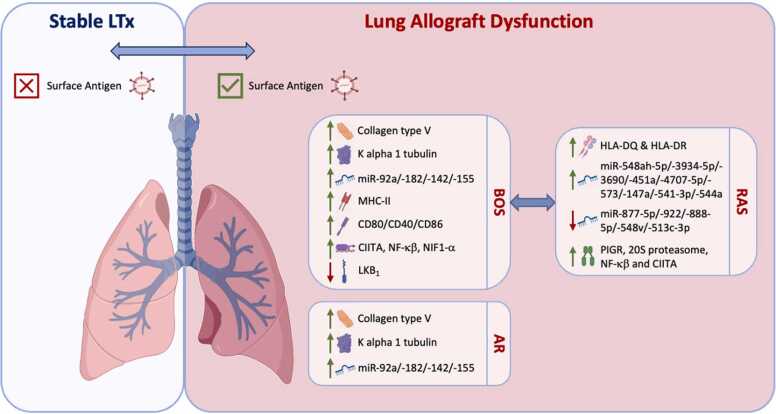
Pathogenic and immunogenic role of EVs in lung allograft dysfunction. Comparing EVs from stable LTx and lung allograft dysfunction patients, those with BOS, RAS and AR demonstrate EVs with SAg, while the EVs from stable LTxR do not. BOS and AR EVs contain increased SAg Col-V and Ka1T compared to stable LTxRs, and elevated expression of miR-92a, miR-182, miR-142–5p and miR-155. EVs from BOS LTxR express MHC-II and co-stimulatory molecules (CD40/CD80/CD86), as well as transcription factors CIITA, NF-κβ and NIF1-α, while levels of LKB_1_ are reduced. Comparing RAS to BOS phenotypes, RAS EVs demonstrate higher HLA-DQ and HLA-DR, higher PIGR, 20S proteasome, NFkB and elevated expression of miR-548ah-5p, miR-3934–5p, miR-3690, miR-451a, miR-4707–5p, miR-573, miR-147a, miR-541–3p, and miR-544a compared to BOS EVs. Expression of miR-877–5p, miR-922, miR-888–5p, miR-548v, and miR-513c-3p is lower in RAS compared to BOS EVs. LTx = Lung transplant; MHC-II = major histocompatibility complex class II; CIITA = class II major histocompatibility complex transactivator; NF-κβ = Nuclear factor kappa-light-chain-enhancer of activated B cells; HLA = Human Leukocyte Antigen; LKB_1_ = liver kinase B1.

Gunasekaran *et al* further investigated whether EVs are induced during lung allograft rejection, examining their origin and antigenic profile, including their microRNA composition and the presence of self-antigens (SAgs).^14^ Their case-control observational study included 30 bilateral LTxRs, divided into three groups: 10 developed CLAD-BOS, 10 developed AR and 10 remained stable. Serum and BAL fluid were collected at 1, 3, 6 and 12 months post-LTx, and EVs were isolated using precipitation and sucrose-cushion. The study demonstrated that serum and BAL EVs from LTxRs with BOS and AR contained SAg, including Col-V and Kα1T, which were absent in EVs from stable patients (Table 1). These findings suggested that EVs containing SAg may act as a non-invasive biomarker for impending rejection, a hypothesis later validated (Col-V and Kα1T) in independent studies (Figure 1) (Table 1).^15^, ^16^ Furthermore, serial sampling revealed that EVs containing Col-V were present in AR and BOS LTxR sera prior to clinical diagnosis of acute rejection and CLAD, further supporting their biomarker potential for early detection.^14^ Interestingly, independent studies have also shown that EV Col-V and Kα1T expression is higher in LTxRs who developed *de novo* donor-specific antibodies (DSA) compared to DSA-negative patients^15^, ^16^ and similarly, only patients with symptomatic respiratory virus infection (RVI), and not stable patients, expressed Col-V and Kα1T^16^ (Table 1). A lack of SAg in stable LTxR also suggested that the detected EVs derived from the transplanted organ, a hypothesis further supported by the finding that donor HLA, but not recipient HLA, was expressed on the surface of EVs.^14^ To investigate the potential role of EV composition in the immunopathogenesis of AR and CLAD-BOS, the authors also profiled the microRNA content of serum-derived EVs. They identified 123 microRNA, of which 15 were higher and 108 were lower in AR compared to stable LTxRs, while 44 were higher and 79 were lower in BOS compared to stable LTxR. MicroRNA target pathway analysis revealed several enriched pathways in AR and CLAD-BOS LTxRs, including PI3K-Akt, Wnt and TGF-β. Specific microRNAs associated with inflammation, endothelial activation, antibody-mediated chronic rejection, and Th17 differentiation (miR-92a, miR-182, miR-142–5p and miR-155) were expressed at higher levels in AR and CLAD-BOS patients compared to stable LTxR (Figure 1) (Table 1).^14^

In two follow-up studies, the same group further explored their hypothesis that circulating EVs activate and sustain immune responses, leading to post-LTx CLAD-BOS. The team examined circulatory EVs isolated from the serum of adult (n=10)^17^ and pediatric (n=6)^18^ LTxR with CLAD- BOS, compared to EVs isolated from stable LTxR (adult n=10, pediatric n=13). Their results confirmed that EVs from CLAD-BOS patients exhibited elevated levels of SAg, including Col-V and Kα1T, relative to stable LTxR. In addition, they identified expression of MHC-II and co-stimulatory molecules (CD40/CD80/CD86) to be a feature of CLAD_BOS-derived EVs,^17^, ^18^ which has been similarly shown by others (Figure 1) (Table 1).^16^ Unique transcription factors were elevated in CLAD-BOS EVs, including immune activating CIITA, NF-κβ, HIF1-α and proteasome 20S (Figure 1).^17^, ^18^ Together, these results support the theory that sustained EV release from LTxR with CLAD-BOS can perpetuate the immune response and stimulate T-cell responses by direct, semi-direct and indirect pathways of Ag presentation.^17^, ^18^ To further explore the pathogenesis of LTx derived EVs, C57BL/6 mice were immunized with 100 μg of pooled EVs isolated from the serum of BOS or stable LTxR EVs at three separate timepoints (between days 1 – 20).^17^, ^18^ In agreement with their previous findings,^14^ the authors observed that mice injected with EVs from CLAD-BOS LTxR demonstrated increased serum Ab to HLA class I,^17^ Col-V and Kα1T^17^, ^18^ and displayed strong humoral immune responses. Furthermore, splenic lymphocytes from CLAD-BOS LTxR injected mice showed increased inflammatory cytokines (IFN-γ and IL-17 in adult models^17^; IFN-γ, TNFα and IL-4 in pediatric models^18^), but decreased IL-10 compared to mice immunized with stable LTxR,^17^, ^18^ suggesting that EVs containing SAg alongside additional cargo can induce and sustain Th17 responses that contribute to BOS^17^ (Table 1). Immunization with pediatric derived EVs from BOS LTxR also resulted in severe lung inflammation and increased fibrosis.^18^

Subsequently, the same group assessed whether the lung SAgs that they identified on CLAD-BOS LTxR EVs had the potential to act as biomarkers for BOS development.^19^ Circulatory plasma EVs were isolated by ultracentrifugation at CLAD-BOS diagnosis, six and twelve months prior to diagnosis (n=41), and from stable controls (n=30), and assessed for EV SAgs Kα1T and Col-V. The authors confirmed elevated Col-V and Kα1T in CLAD-BOS LTxRs compared to stable recipients at the time of CLAD-BOS diagnosis and furthermore, LTxR EVs demonstrated higher levels of Kα1T and Col-V at 6 and 12 months prior to CLAD-BOS diagnosis. These results were validated in two independent cohorts,^19^ in patients with CLAD,^20^ and a separate cohort of pediatric patients,^18^ suggesting a role for circulating EVs as a biomarker for prevention or early treatment of rejection following LTx (Figure 1) (Table 1).

Liver Kinase B1 (LKB1), a tumor suppressor gene, has also been associated with EV-exacerbated CLAD, via regulation of epithelial-mesenchymal transition (EMT).^20^, ^21^ Specifically, LTxR demonstrated significantly lower levels of LKB1 at both the mRNA and protein levels in circulating EVs isolated from LTxR diagnosed with primary graft dysfunction (PGD), compared to no-PGB^21^ (Figure 1) (Table 1). This was confirmed in an independent study focussed on plasma EVs isolated from patients with CLAD, and observed 12 months prior to CLAD diagnosis, indicating reduced LKB1 in circulating EVs as a potential biomarker for CLAD risk^20^ (Table 1). The authors hypothesized that reduced LKB1 leads to EMT and fibrosis of small airways, key features of CLAD after LTx.

Bansal *et al* further assessed EV immunogenic properties by examining differences in plasma EVs from LTxR with CLAD-BOS (n=18), CLAD-RAS (n=13), or stable LTxR (n=5), focusing on immunological markers and microRNA expression.^22^ The authors showed significantly higher levels of HLA-DQ and HLA-DR in RAS compared to BOS EVs (Figure 1), suggesting that EVs with surface HLA class II molecules may induce persistent immune responses in RAS-phenotype CLAD.^22^ CLAD-RAS EVs also showed higher levels of PIGR, 20S proteasome, NF-κβ and CIITA (Figure 1), further indicating EVs from CLAD-RAS LTxRs to be highly immunogenic, resulting in humoral immunity to mismatched HLA. The group also assessed microRNA expression and identified n=14 miRNAs with significant expression differences between CLAD-RAS and CLAD-BOS EVs, of which nine were higher in CLAD-RAS EVs (miR-548ah-5p, miR-3934–5p, miR-3690, miR-451a, miR-4707–5p, miR-573, miR-147a, miR-541–3p, and miR-544a) and five were higher in CLAD-BOS EVs (miR-877–5p, miR-922, miR-888–5p, miR-548v, and miR-513c-3p) (Figure 1) (Table 1).^22^ Target analysis demonstrated multiple pathways, including TGF-β signaling. A more recent focus on microRNA biomarkers for CLAD outcome identified higher levels of plasma EV miR-17–5p and miR-150–5p expression in patients who developed CLAD (n=59) compared to those who remained CLAD-free.^23^ This further suggests the potential of EV microRNAs as biomarkers of CLAD outcome.

Overall, studies assessing EVs in the context of LTx suggest their immunogenic role, whereby sustained release of EVs from the transplanted organ contributes to the pathogenesis of chronic rejection via humoral and cellular immune responses. Comparing BOS to RAS CLAD phenotypes, CLAD-RAS EVs demonstrate a higher concentration of proinflammatory factors, HLA class II, lung SAgs and antibodies to HLA Class II molecules, indicating severe allograft injury.

### Extracorporeal photopheresis therapy for CLAD

Accumulating data suggests that the use of extracorporeal photopheresis (ECP), an immunomodulating therapy, can slow the course of CLAD in some patients.^24^ ECP is a three-step procedure that comprises of; separation of leukocytes from peripheral blood during a leukapheresis procedure, photoactivation of the collected cells, and reinfusion of treated cells back to the patient. To facilitate the leukapheresis step, an intravenous line is inserted to collect whole blood from the patient. Red cells and plasma are returned to the patient, while the leukocytes are treated with a photosensitizing agent Methoxsalen (8-methoxypsoralen), followed by UVA irradiation exposure (320–400 m wavelength). This causes cross-linking of DNA within the nuclei of lymphocytes, leading to apoptosis of 5–15% of treated cells.^25^ The pre-apoptotic cells are then returned to the patient, where they exert undefined immunomodulatory effects.

Early studies suggested ECP’s beneficial mechanism of action (MoA) to be driven by leukocyte apoptosis, however, it is now appreciated that a complex immunomodulatory approach is at play. This includes the initiation of dendritic cells (DC), switch in antigen presenting cell (APC) activity, modification of cytokine profiles, and stimulation of T-cell lineages, including T regulatory cells (Tregs), which has been extensively reviewed elsewhere.^25^, ^26^, ^27^ In LTx recipients (LTxR) that reach functional stabilization, ECP increases or stabilizes the number of CD4^+^CD25^+^FoxP3^+^ Treg-cell counts, mediates suppression of humoral and cellular rejection and induces tolerance, thus prolonging survival of the transplanted organ.^28^

Studies assessing the effect of ECP in LTxR with CLAD have shown promise in slowing or halting CLAD progression^29^ (Table 1). Yet, the molecular mechanisms driving ECPs immunomodulatory action, whilst preserving immune function, are poorly understood. To date, there is no clear mechanistic understanding of how ECP treated immune cells that undergo apoptosis after re-infusion into the patient exert an immunomodulatory effect on the rest of the immune system. However, we believe cell to cell communication via circulating mechanisms may be an important contributing factor.

### Extracellular vesicles and their effect on extracorporeal photopheresis mechanism of action

Although the precise mechanisms of action of ECP remain incompletely defined, EVs have emerged as potential mediators of its immunomodulatory effects. In particular, though alteration of APC function and cytokine and microRNA signaling networks, thereby contributing to the broader immunoregulatory milieu induced by ECP.

EV-associated microRNAs represent a critical mechanism contributing to ECP-mediated immune modulation. MicroRNA-155, which has been shown to be upregulated in ECP responders,^30^ may have dual roles in this context. Under pro-inflammatory conditions, it promotes DC maturation and M1 macrophage polarization,^31^ but it is also involved in Treg development and immune tolerance.^32^ Similarly, miR-146a, which is also increased following ECP treatment,^30^ negatively regulates innate immunity by targeting TRAF6 and IRAK1/2, thus reducing NF-kB activation and pro-inflammatory cytokine production by macrophages and DCs.^33^ In addition to effects on myeloid cells, EV-associated microRNA have been implicated in modulating natural killer (NK) cell activity, through regulation of activating and inhibitory receptor expression, thus shifting NK cytotoxicity.^34^ These findings suggest that EVs contribute to a complex immunoregulatory environment that extends beyond DCs and T cells, reinforcing the tolerogenic impact of ECP. Despite these insights, mechanistic studies specifically addressing the role of EVs in coordinated immune cell interactions during ECP immunomodulation are extremely limited, particularly in the context of lung allograft dysfunction.

Recently, it has been shown that platelets release EVs that are processed through ECP and subsequently transfused back into the patient, delivering potent immunomodulation. MacLeod *et al* assessed the effects of exposing platelets and their EVs directly to UVA/8-methoxypsoralen by isolating platelet-rich plasma (PRP) from healthy donors and measuring platelet activation and aggregation following UVA/8-methoxypsoralen exposure.^35^ Interestingly, they observed no difference in platelet aggregation, marker activation, soluble P-selectin or platelet factor 4 levels, nor any changes in EV size or concentration following UVA light or 8-methoxypsoralen exposure alone/in combination.^35^ This suggests that according to in vitro studies, platelet-derived EVs may not significantly contribute to the mechanism of ECP action. However, the effect of mechanical forces and platelet-leukocyte interactions that occur during the in vivo ECP procedure remain unknown.

**Figure 2:**
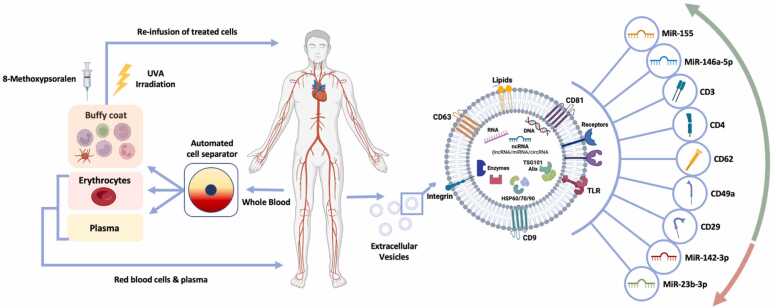
Effect of ECP on EVs following treatment for CLAD. During ECP, whole blood is drawn from the patient, and separated by apheresis into the buffy coat, erythrocytes and plasma. The erythrocytes and plasma are immediately returned to the patient, while the leukocytes are treated with 8—methoxypsoralen and exposed to UVA irradiation. The treated cells are then returned to the patient. Extracellular vesicles isolated from patients undergoing ECP have been shown to have elevated levels of miR-155 and miR-146a-5p, CD3, CD4, CD62, CD49a and CD29 expression, while levels of miR-142–3p and miR-23b-3p were decreased. Figure partially amended from.^37^

In a pilot clinical trial conducted by Rosso *et al*,^36^ n=24 patients receiving ECP following LTx for cystic fibrosis were assessed for EV characteristics and surface markers. The group observed that patients receiving ECP demonstrated upregulation of platelet (CD62), lymphocyte (CD3, CD24) and integrin (CD29, CD49e) derived EV markers, compared to controls (Figure 2). Although the results were preliminary, the authors concluded that specific EV antigen signatures may represent a promising approach to better understand the immunomodulatory effects of ECP, both at a molecular and cellular level.^37^

To further explore the molecular MoA of ECP, Bozzini *et al* explored microRNA expression differences during CLAD response to ECP.^38^ This comparative pilot study focussed on serum from n=26 patients undergoing ECP for BOS, comprising a mix of off-line and closed system methods, and n=16 gender and age matched healthy controls. The authors assessed 14 microRNAs previously associated with the proliferation and function of immune cells of innate and adaptive immunity, by measuring their expression at ECP baseline and 6-month timepoints. They observed significant downregulation of miR-155–5p, miR-146a-5p and miR-31–5p in LTx patients at ECP baseline compared to controls. After 6 months of ECP, expression of miR-155–5p was increased compared to baseline, while no significant difference in miR-146a nor miR-31–5p expression was observed (Figure 2). In addition, expression of miR-23b-3p was reduced by ECP, to levels comparable to healthy controls (Figure 2). There was no difference in microRNA expression in ECP responders vs. non-responders. However, when limited to the responder group only, there was a significant increase in miR-155–5p and decrease in miR-23b-3p at 6 months compared to baseline (Figure 2). Target enrichment analysis revealed SMAD4 to be a common microRNA target, and its expression was downregulated in PBMC isolated from the same patients at 6 months. Conversely, miR-155 expression was elevated in PBMC at the same time point. This suggests that miR-155 may be targeting SMAD4, which has a role in TGF-B mediated immunoregulation and fibrogenesis. The authors concluded that a specific miRNA signature may be associated with functional stabilization or improvement following ECP, with miR-155 and miR-23b showing promise for identifying ECP response.^38^ Although the study was based on small numbers, results suggested that further longitudinal clinical and mechanistic studies to assess microRNAs in ECP immunomodulation are warranted.

In a subsequent follow-up study, Bozzini *et al*^30^ performed a prospective pilot in vitro study to assess whether ECP effects on miRNA and cytokine dysregulation are due to the modulation of mononuclear cell (MNC) activation and function. The study included n=16 adult patients with BOS who were treated with ECP using the off-line method. Plasma samples and MNCs were collected at ECP enrollment and after 10 cycles of treatments. The authors assessed the expression of miRNAs previously associated with BOS and/or ECP response,^38^ as well as microRNAs involved in the regulation of DC, B and T cell immunosuppressive properties. After 10 cycles of ECP, EV miR-142–3p expression was down-regulated compared to pre-treatment levels, while miR-146a-5p expression was up-regulated by ECP (Figure 2). MiR-142–3p has been previously shown to modulate Treg function, whereby its down-regulation confers suppressor functions to Treg cells.^39^ MiR-146a has a role in the negative regulation of immune response, specifically, of myeloid cells via TNF receptor-associated factor 6 (TRAF6) and interleukin receptor-associated kinase 1/2 (IRAK1/2).^33^, ^40^ MiR-146a is also reported to be elevated in Tregs and induced upon activation,^41^ increased in monocytes in response to TLR4 stimulation,^33^ and involved in the survival and TLR-induced maturation of pDCs.^42^ The authors inferred that ECP may therefore affect Treg cell function, since both miR-142 and miR-146a have been shown to regulate suppressor Treg function and DCs. They concluded that EV microRNAs may be implicated in the ECP MoA and warrant further study.^30^

### Future outlook for EV biology in lung transplantation and CLAD

The outlook for research on EVs in LTx and CLAD is both promising and challenging. As EVs continue to emerge as key players in immunomodulation, antigen presentation, and the progression of allograft dysfunction, there is a clear need for further studies to refine our understanding of their role in CLAD development. Additionally, exploring their contribution to the mechanisms of immunomodulatory therapies, such as ECP, and their potential as translatable biomarkers for early CLAD detection and prognosis, offers significant promise. These advancements could lead to more personalized diagnostic tools, ultimately improving patient management and treatment outcomes.

Future research priorities should focus on several key areas. Firstly, mechanistic studies are needed to elucidate the molecular interactions between EVs and immune cells in the context of CLAD pathogenesis, including the dynamics of EV release during the rejection progression, as well as comprehensive characterization of EV cargo beyond the currently identified self-antigens and microRNAs. Understanding how different EV subpopulations may contribute to pathogenesis of BOS versus RAS phenotypes will be crucial for targeted interventions. Secondly, Investigating the roles of EVs during ECP immunomodulation is a critical area of unmet need. Such studies should focus on how photopheresis alters EV composition and function in ECP-treated cells, whether such cells release EVs with distinct immunoregulatory properties, and how EVs might mediate tolerance induction and Treg expansion during treatment. Thirdly, clinical translation of EV-based biomarkers requires standardization of isolation, characterization and analysis methods, as well as extensive validation in larger, independent, longitudinal cohorts. Future research may establish EV properties as part of risk stratification models, to guide personalized treatment strategies and identify patients that may benefit from prophylactic interventions.

Given the complexity of CLAD pathogenesis, ECP immunomodulation and EV-mediated immune crosstalk, a multidisciplinary approach is crucial. This should incorporate immunology, EV biology, transplant medicine, molecular biology, genomics and proteomics. Furthermore, this needs to be integrated into sophisticated translational research study designs, preferentially conducted alongside well-powered, prospective, randomized and multi-center clinical trials to evaluate the efficacy of ECP and its potential for earlier or prophylactic intervention. Ultimately, the end goal is to improve patient outcomes and extend graft survival for lung transplant recipients through the development of comprehensive monitoring systems that enable precision medicine approaches to transplantation.

## Funding & Acknowledgments

REC is supported by an International Society for Heart and Lung Transplantation (ISHLT) Extracorporeal Photopheresis Immunomodulation in Thoracic Transplantation Challenge Grant, supported by Mallinckrodt Pharmaceuticals, and a research grant from the UK Photopheresis Society. SJB is supported by a PhD Studentship from The Bubble Foundation. AF is supported by the National Institute for Health and Care Research (NIHR) Blood and Transplant Research Unit in Organ Donation and Transplantation (NIHR203332), a partnership between NHS Blood and Transplant, University of Cambridge and Newcastle University. The views expressed are those of the author(s) and not necessarily those of the NIHR, NHS Blood and Transplant or the Department of Health and Social Care.

## Disclosure

The authors of this manuscript have no conflicts of interest to disclose.

## Declaration of Competing Interest

The authors declare that they have no known competing financial interests or personal relationships that could have appeared to influence the work reported in this paper.
